# Mitigating the Blurring Effect of CryoEM Averaging on a Flexible and Highly Symmetric Protein Complex through Sub-Particle Reconstruction

**DOI:** 10.3390/ijms25115665

**Published:** 2024-05-23

**Authors:** Diana S. Suder, Shane Gonen

**Affiliations:** Department of Molecular Biology and Biochemistry, University of California Irvine, Irvine, CA 92697, USA

**Keywords:** CryoEM, protein design, single-particle, cage, scaffold, sub-particle, flexibility

## Abstract

Many macromolecules are inherently flexible as a feature of their structure and function. During single-particle CryoEM processing, flexible protein regions can be detrimental to high-resolution reconstruction as signals from thousands of particles are averaged together. This “blurring” effect can be difficult to overcome and is possibly more pronounced when averaging highly symmetric complexes. Approaches to mitigating flexibility during CryoEM processing are becoming increasingly critical as the technique advances and is applied to more dynamic proteins and complexes. Here, we detail the use of sub-particle averaging and signal subtraction techniques to precisely target and resolve flexible DARPin protein attachments on a designed tetrahedrally symmetric protein scaffold called DARP14. Particles are first aligned as full complexes, and then the symmetry is reduced by alignment and focused refinement of the constituent subunits. The final reconstructions we obtained were vastly improved over the fully symmetric reconstructions, with observable secondary structure and side-chain placement. Additionally, we were also able to reconstruct the core region of the scaffold to 2.7 Å. The data processing protocol outlined here is applicable to other dynamic and symmetric protein complexes, and our improved maps could allow for new structure-guided variant designs of DARP14.

## 1. Introduction

Over the last decade, single-particle cryogenic electron microscopy (CryoEM) has become a powerful structural biology method routinely used to study proteins at high resolution [1]. As instrumentation and processing software improve, increasingly higher resolutions are achievable [2], less starting material is required [3,4], and the technique can be applied to ever more complex and difficult targets [5]. As images of individual proteins are used, different conformational states can also be observed by separation and classification during data processing [6,7]. However, major challenges deterring high resolution studies still exist, including protein size [8] and dynamics [9]. Small proteins of ~50 kilodaltons (kDa) or smaller are difficult or sometimes impossible to study on their own [8], and flexible protein regions can blur out high-resolution information [10,11].

CryoEM reconstructions rely on aligning and averaging images of thousands of individual proteins. The overall movement of proteins in ice during data collection is typically alleviated by motion correction, where individual movie frames are realigned [12,13]. However, dynamics inherent to the proteins themselves are still a barrier to high resolution due to averaging [14]. This issue can be even more pronounced when symmetry-related subunits are involved [15]. Many different strategies have been described to try to mitigate these issues, including biochemical stabilizers [16,17], binding of ligands aiming to lock the protein into one conformation [18], and fiducial markers for alignment [19]. Additionally, many data processing methods have also been developed to address this issue, including focused refinement, whereby small areas of the map are used as targets while others are masked or subtracted away from the data [20]; non-uniform refinement [10]; symmetric expansion [21]; multi-body refinement [22]; and sub-particle reconstruction [23], whereby each individual subunit making up the complex is isolated and averaged as a separate particle. Recently, computationally designed proteins called scaffolds, large proteins designed to bind and display small proteins of interest for high-resolution reconstructions, have been developed [24,25,26]. While these scaffolds aim to bypass the protein size barrier, flexibility of the scaffold architecture and/or the binding of target proteins can still exist, limiting high-resolution detail.

**Figure 1:**
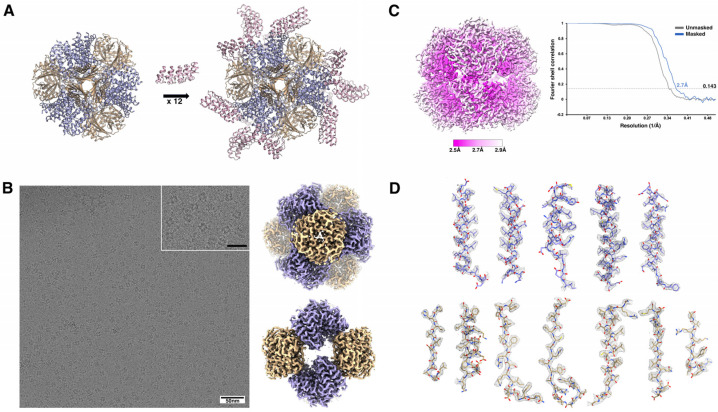
Design overview of DARP14 and CryoEM map of the core scaffold. (**A**) A tetrahedrally symmetric protein scaffold was designed [25] to incorporate small proteins called DARPins via helical extensions in each chain of each subunit A. Subunit A now features three DARPins, totaling 12 DARPins displayed on the protein scaffold, together termed DARP14. (**B**) (**left**) CryoEM micrograph of DARP14 (inset—zoomed-in view, scale bar = 25 nm). (**right**) 2.7 Å map of the core tetrahedral scaffold of DARP14, viewed along the 3- and 2-fold axes, with DARPins masked out. (**C**) (**left**) Local resolution of the map from (**B**) with the corresponding Fourier Shell Correlation (**right**). (**D**) Our refined atomic model was fit into the EM map from (**B**) using UCSF ChimeraX (v1.5) with the “fit in map” command and displayed using the mesh map view. Trimeric A subunits in purple, B subunits in yellow, and DARPin subunits in pink.

Here, we describe and detail the use of sub-particle CryoEM averaging and present updated reconstructions of DARP14, a multi-subunit tetrahedrally symmetric protein complex. Originally described by Liu and Gonen et al. (2018) [25], DARP14 was computationally designed as a scaffold capable of displaying small proteins for CryoEM (Figure 1A). DARPins are small, ~17 kDa helical repeat proteins that feature variable residue loops capable of selective non-covalent binding to other proteins [27], akin to antibodies. Using helical extension, DARPins were genetically fused to one of two trimeric subunits making up a previously designed, self-assembling cage called T33-21 [28]. Since T33-21 assembles with 4 copies of each trimeric subunit (A and B), 12 copies of DARPin are displayed on the final 24-subunit, ~600 kDa chimera termed DARP14 (Figure 1A). Variants of DARP14 and similar scaffolds have since been generated and used to bind and image small proteins, such as GFP [29] and KRAS [30]. In the original 2018 study of DARP14, the core of the protein making up the original T33-21 design was found to be very stable and could be resolved to 3.2 Å resolution when applying tetrahedral symmetry during averaging. In contrast, the DARPin proteins were found to have a marked deviation from the original designed helical extension. Owing to their flexibility away from ideal tetrahedral symmetry and possible terminal unwinding, they could not be resolved to the same resolution ranges as the core and could only be observed at much lower resolution ranges.

The methodology we outline here resulted in new reconstructions of DARP14, improving on the original core T33-21 protein to 2.7 Å resolution (Figure 1B–D), and, crucially, significantly improves the DARPin reconstruction. Using new software and various refinement methods, mainly sub-particle reconstruction, our new maps allowed us to accurately observe the DARPin–cage helical extension as well as areas of close contact between the two proteins. We describe our structural data processing approach, detail the new maps, and discuss their observable features. The approach used here should be applicable to the refinement of most flexible proteins, including highly symmetric proteins and newly designed variants of DARP14.

## 2. Results and Discussion

**Figure 2:**
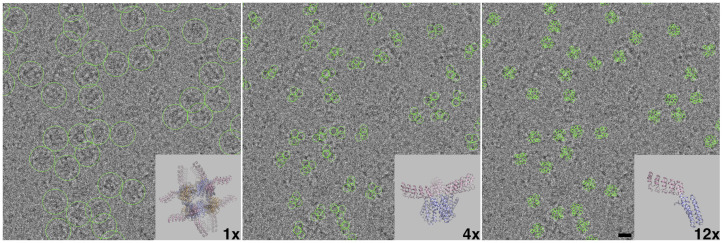
Sub-particle reconstruction overview. (**Left**) A representative section of a micrograph with many copies of the full tetrahedral DARP14 protein, circled. (**Center**) The same representative micrograph with C3 symmetrical trimer coordinates circled; each full DARP14 protein is composed of four trimers. (**Right**) The same representative micrograph with individual DARPin coordinates circled; each full DARP14 protein can result in up to 12 monomeric DARPin coordinates. Scale bar = 10 nm. These micrographs visually represent how one DARP14 cage ultimately gave rise to signal for 12 DARPins that could be combined during data processing to mitigate the effect of symmetry of this small flexible subunit.

**Figure 3:**
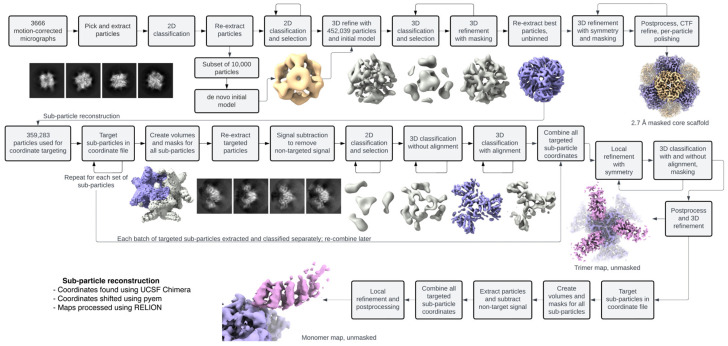
Data processing overview. The CryoEM processing pipeline used in this study is outlined using the sub-particle averaging approach to reach the final trimeric and monomeric maps. The software required for the protocol used here of sub-particle reconstruction is outlined.

While symmetry can be very beneficial for CryoEM analysis, essentially multiplying the number of particles, for some proteins where dynamics are present, symmetry can have a multiplicative negative effect. The processing pipeline we chose leveraged symmetry at the low-resolution level (initial alignment and three-dimensional (3D) refinement) and mitigated the detrimental blurring effects of symmetry when accuracy was required at high resolution (focused refinement of the flexible DARPins). Sub-particle reconstruction and our processing pipelines are summarized in Figure 2 and Figure 3. High-resolution reconstructions of DARP14 can be calculated using standard methods of CryoEM, and medium- to low-resolution maps only require a few thousand particles. These reconstructions typically have strong signal for the core cage, but the DARPins, due to their flexible nature, are not prominent. For the DARPin signal, we turned to sub-particle reconstruction coupled with signal subtraction. These two processing methods rely on accurate alignment at high resolution so that only the correct signal is subtracted, and particle coordinates can be shifted accurately to the corresponding protein sections for focused refinement. We therefore first reconstructed the full DARP14 complex to an overall resolution of 3.1 Å. For this initial reconstruction, particle counts were favored over slight improvements to resolution to keep as much data intact for future rounds of sub-particle analysis and classifications. This reconstruction therefore served as a foundational starting point for all subsequent sub-particle refinements.

In addition to focusing on the DARPin components, we also set out to extract as much high-resolution information as possible from the stable core cage component, this time by masking out the dynamic parts of the complex. Using available and established processing software and scripts combined with careful 3D classifications, signal subtractions, and sub-particle reconstructions, we determined CryoEM maps including the core cage component and the isolated symmetric pieces of DARP14 containing trimeric and monomeric DARPins.

### 2.1. Sub-Particle Reconstruction of Trimeric and Monomeric Subunits of DARP14

**Figure 4:**
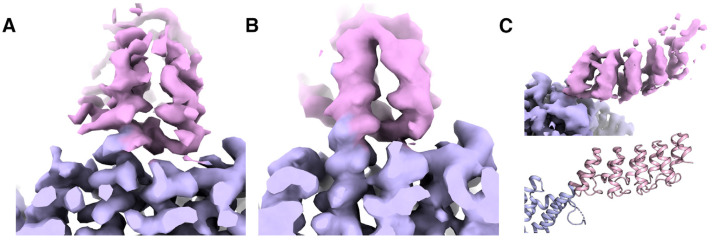
Trimeric and monomeric DARPin CryoEM maps obtained through sub-particle reconstruction and subtraction. (**A**) Trimeric map showing the designed helical extension into the DARPin. Clear secondary and side-chain information can be observed. (**B**,**C**) Monomeric map showing the helical extension view (**B**) and side view, where the helical repeats can be observed and oriented to the model (**C**).

For sub-particle reconstruction and signal subtraction, ideally only the components of interest (in this case each DARPin trimer or monomer) would be the sole focus. However, since these components are very small, they are still difficult to align during refinement. Therefore, for both the trimeric and subsequent monomeric reconstructions, extended parts of the core cage were kept intact to allow for sufficient signal to remain for classifications and local refinement. Coordinates were shifted from the main cage to the center of each of the four trimeric A subunits, followed by masking and subtraction (Figure 2 and Figure 3). These sets were then combined, quadrupling the initial particle count. We were then able to proceed with further classifications and refine the particles using applied C3 symmetry. An example 2D classification is shown in Figure S1, whereby high-resolution features for DARPin were chosen for the next steps. Using the newly calculated C3 symmetric map, the DARPins were more defined at a higher overall resolution than the full tetrahedrally symmetric DARP14 map, with observable secondary structure and some side-chain features (Figure 4A).

Stemming from the previous trimeric particle dataset, further subtraction and sub-particle reconstructions were made whereby each monomeric subunit was isolated. Using the same methodology as in the preceding run, the coordinates were shifted from the trimers to the center of each of the three monomers and combined, tripling the dataset. At this point, up to 12 DARPin monomers were retained from each full DARP14 particle found in the micrographs (Figure 2). Since the monomers lack symmetry, our final reconstructions were therefore made using no applied (C1) symmetry.

In contrast to the full complex, the resulting trimeric and monomeric DARPin maps feature vastly improved high-resolution information, particularly for the helical extension linker and residues close to the cage, facilitating model building and demonstrating the effectiveness of reducing the effect of high symmetry on averaging (Figure 4). The most significant improvement to the map came from reducing T-symmetry to C3. The resolution range for both C3 and C1 was comparable; however, C3 DARPin information looked clearer closer to the core cage, while C1 DARPin looked clearer further away from the core cage. The latter residues of the DARPins remained at lower resolutions, likely owing to a significantly higher degree of flexibility in those regions and possible unwinding of the structure [25]. A significantly higher number of particles will need to be collected to attempt further classifications to see if signal can be improved at those locations.

### 2.2. Stabilizing the DARPin–Cage Interaction

All DARPin proteins displayed on the scaffold contain a single anchoring point, the extended helical linker. While further interactions between neighboring DARPins have proved to help stabilize a variant of the complex [30], additional interactions could be made using structure-guided design between the core cage and DARPin. Several residues can be found in close proximity to the core cage, potentially allowing for non-covalent interactions and/or covalent disulfide bridges. A secondary anchor point could stabilize the movement of the entire DARPin in relation to the core cage, allowing for symmetrical reconstruction and refinement and eliminating or reducing the need for focused refinements. Additionally, results from the original 2018 manuscript [25] discovered a deviation of the helical extension away from the design. It may be possible to further strengthen the helical extension without modifying the cage by finding residues amenable to mutations compatible with cystine cross-linkers for intra-residue interactions [31].

### 2.3. Reconstruction of the Core Cage of DARP14

To push for the highest resolution possible given the dataset, we created a mask that excluded the flexible DARPin components while keeping the core cage of DARP14 intact. Multiple rounds of 3D classifications and refinements using tetrahedral symmetry were made until no improvements to the map were observed. An example 2D classification is shown in Figure S2, whereby high-resolution features were chosen for the next steps. A final set of 277,091 particles were postprocessed using multiple rounds of Bayesian polishing and CTF refinement to obtain a final map of the core cage at 2.7 Å (Figure 1B–D and Figure 3). Local resolution is shown in (Figure 1C). This is a marked improvement in overall resolution compared to the original 3.2 Å study. At this resolution, we could model nearly every side chain of the complex. The map also shows possible sites of ions (mainly in the 3-fold trimeric interfaces) and water molecules (not modeled). The previous DARPin core model (PDB ID 6C9I) was initially fit into our new map using UCSF Chimera (v1.15) with the “fit in map” command and refined using Phenix v1.20.1 and COOT v0.9.5 (see Materials and Methods) and displayed using UCSF ChimeraX (v1.5) (Figure 1D). At this point the resolution of the core cage is likely reaching the limits inherent to the sample and data collection parameters.

### 2.4. Processing Highly Symmetric Protein Complexes by CryoEM

Sub-particle reconstruction can be combined with new advances and methods of CryoEM processing to extract information from other highly symmetric and non-symmetric particles, even those with minimal flexibility. The tools for sub-particle reconstruction are readily available; however, studies and protocols for using it are currently sparse. Here, we described the processing pipeline when applied to a tetrahedrally symmetric scaffold protein DARP14. Our resulting maps could allow for structure-guided redesign of the original DARPin construct.

## 3. Materials and Methods

### 3.1. CryoEM Data Processing

The previous dataset of 3666 movies was imported into RELION 3.0.8 [32]. Raw movies were corrected for beam-induced motion using the RELION implementation algorithm and all 24 movie frames. CTF estimation was carried out using the GCTF [33] executable in RELION. Particle coordinates were picked automatically with optimized parameters using Gautomatch v0.53 [34] and imported into RELION, then extracted and binned to speed up processing. All subsequent steps were performed in RELION unless otherwise specified. Reference-free 2D classification was carried out initially using 200 classes, and those displaying recognizable features were selected. Selected particles were re-extracted and re-centered. To generate a C1 (non-symmetric) de novo initial model, a subset of these particles having already undergone one round of selection were subjected to another 2D classification, this time using 100 classes. The classes with the strongest and clearest signal were selected and limited to 10,000 particles as the input for initial model creation. An additional initial model was made from the C1 model by applying T-symmetry with relion_image_handler. The full set of selected classified particles was re-extracted and split into three groups of approximately 500,000 particles each to speed up processing and enable finer sampling, then subjected to two more rounds of 2D classification. Classes displaying recognizable particles were selected with increasingly stringent criteria each time; selected particles were eventually re-extracted with a box size of 192 pixels, binned by two (1.31 Å/pixel), and joined into one set of 452,039 particles to continue to 3D processing.

All 452,039 particles were refined without applied symmetry and without a mask. This refinement provided an EM map displaying at least partial signal for most of the 12 DARPins on the full scaffold, albeit at a low resolution.

### 3.2. Refinement of the Core Scaffold to 2.7 Å Resolution

To refine the core scaffold to the highest possible resolution, we first created a solvent mask that excluded the flexible DARPins using UCSF Chimera v1.15 [35]. Starting from the first refinement, we ran a 3D classification with 4 classes in C1. The best class with 359,283 particles was selected as the input into a second 3D refinement. This process of 3D classification followed by class selection and 3D refinement was repeated twice, keeping the best particles. An example 3D classification is highlighted in Figure S3, whereby the highest resolution class was chosen for the next steps of processing. A selected class of 309,973 particles was autorefined in T symmetry, then subjected to a round of postprocessing, CTF refinement, Bayesian polishing, and 3D refinement. Then, the particles were 3D classified again without angular sampling, with the intent of keeping the most well-aligned particles. Selected particles were again refined, postprocessed, CTF refined, Bayesian polished, refined, and again subjected to 3D classification where the best class of 277,091 particles was selected. These remaining 277,091 particles were subjected to autorefinement, postprocessing, CTF refinement, and Bayesian polishing. They were re-extracted to a pixel size of 0.9825 Å/pixel and a box size of 296 pixels. The final refined and postprocessed reconstruction containing only the core cage component of DARP14 reached an overall resolution of 2.69 Å using the gold-standard FSC 0.143 threshold.

### 3.3. Sub-Particle Reconstruction of the Trimeric Subunits of DARP14

As a starting point for breaking up the components of DARP14 into their trimeric A subunits using sub-particle reconstruction, an early refinement of the full DARP14 particle was used (the same as for the previous section), composed of 359,283 particles. Using UCSF Chimera v1.15, coordinates in 3D space that corresponded to the center of each of the four A subunit trimers were found. Using the subparticles.py script available in the pyem package v0.5 [36], the coordinates of the refinement star file were shifted to the new trimer coordinates four times, once for each trimer.

Using UCSF Chimera v1.15, two maps and corresponding masks were made for each of the four trimers—one containing the trimer only (volume to keep) and one containing everything else (volume to subtract). Some extended parts of the neighboring subunits were kept intact with each trimer to aid alignment. Using the same parameters as for subparticles.py, the map.py script (pyem package [36]) was used to re-center the .mrc files. All 359,283 particles were then re-extracted four separate times using each shifted .star file with a box size of 240 pixels; the box size was chosen to appropriately frame the trimer. Signal subtraction was performed four separate times using these particles and their corresponding .mrc files and masks, resulting in particles containing a trimer of DARPins and most of the main scaffold removed. Successful subtraction was confirmed using a subset of particles and relion_reconstruct. Similarly, a cut down version of the initial model was made, representing only the relevant signal.

Following signal subtraction, multiple rounds of 2D classifications were performed where strong DARPin signal was favored between rounds. Iterative rounds of maskless 3D classifications with and without alignment followed until the best class converged. An example 3D classification is highlighted in Figure S3, whereby the highest resolution class was chosen for the next steps of processing. Finally, a mask containing the three DARPins was made to isolate the flexible structure of interest and used for new rounds of 3D classifications using C1 (no symmetry). All four trimeric particle groups were joined into a single curated set of 366,069 particles. This curated set was then used to push to the highest resolution and map quality possible with new rounds of 2D and 3D classifications using C1 and C3 symmetry, CTF refinement, particle polishing, and postprocessing. The final map used 95,889 particles refined with C3 symmetry. Local resolution estimates ranged from ~3.3–5 Å across the first two helical repeats and ~5–8 Å towards the tip of the DARPin component.

### 3.4. Sub-Particle Reconstruction of the Monomeric Subunits of DARP14

Using the final trimeric set containing 95,889 particles, a similar protocol was followed as in the previous section to target each individual DARPin, tripling the number of particles in the dataset. Further rounds of 3D classification did not reveal classes that were of sufficiently better resolution or map quality; therefore, the final refinement used all 287,667 particles without symmetry.

### 3.5. Model Building, Refinement, and Validation

For the core scaffold, a previously published set of coordinates, PDB 6C9I, was aligned into the final refined EM map using UCSF Chimera v1.15 [37] with the “fit in map” command, and residues were allowed to be refined individually against the map using Phenix.real_space_refine [38]. The new atomic coordinates from Phenix (v1.20.1) were imported into COOT [39] (v0.9.5) to be manually inspected for steric clashes and other potential issues. For the DARPin monomer, a subset of previously published coordinates (PDB 6C9K) containing only chain A and the attached DARPin were used, along with the final refined EM map for the DARPin component, and were aligned in UCSF Chimera v1.15. Residues were allowed to be refined individually against the map using Phenix.real_space_refine, and the EM map and coordinates were imported into COOT to be manually inspected. MolProbity [40] was used inside of the Phenix software package to evaluate global and local model quality. Model building and validation statistics are available in Table 1; all the values that appear in this table under Model Validation were generated in Phenix v1.20.1 and COOT v0.9.5 during the described procedures.

Figures were prepared using UCSF Chimera v1.15 and v1.16 [35], UCSF ChimeraX v1.3 and v1.5 [37], PyMOL v2.3.0 [41], Microsoft PowerPoint v16.78.3 (Microsoft, Redmond WA, USA) and Adobe Photoshop 2022 (Adobe, San Jose CA, USA).

## 4. Conclusions

Protein flexibility is often detrimental for calculating high-resolution CryoEM maps due to averaging. Here, we detail the use of sub-particle reconstruction combined with signal subtraction when applied to a highly symmetric protein scaffold. Improvement of the map with respect to the flexible DARPin components was observed, with both secondary structure and side-chain information, particularly close to the core cage. Future redesigns of DARP14 informed by our new maps could be made. This methodology can be combined with other processing steps as well as biochemical or construct optimizations to help mitigate issues of flexibility. While we outlined a strategy for reducing the effects of symmetry by realigning the individual building-block subunits, other datasets containing non-symmetric proteins could benefit from this strategy with realignment to focus on areas of flexibility for classifications and local refinements with or without the subtraction steps. Large particle numbers and sufficient signal are still needed for this strategy; however, in the future, new improvements to equipment and data collection strategies should reduce these requirements. As the complexity of proteins studied through CryoEM increases, methods to extract as much information as possible from the data through processing can become a crucial factor in observing important details.

## Figures and Tables

**Table 1:** CryoEM data collection and processing parameters.

Experimental Parameters	Experimental Values	
Imaging		
Grid type	C-Flat 1.2/1.3 200-mesh (EMS)	
Microscope	Titan Krios	
Camera	Gatan K2 direct electron detector	
Voltage	300 kV	
Magnification	22,500×	
Dose per frame	1.2 e^−^/Å^2^	
Frames per movie	24	
Defocus range	−0.7 to −2.5 μm	
Pixel size	0.655 Å/px	
Number of movies	3666	
CryoEM Maps	Core Scaffold	DARPin Trimer/Monomer
Number of particles	277,091	95,889 (Trimer), 287,667 (Monomer)
Pixel size	0.9825 Å/px	1.31 Å/px
Box size (px)	296	240
Overall map resolution	2.7 Å	N/A
FSC for resolution calculation	0.143	0.143
Applied Symmetry	T	C3 (Trimer), C1 (Monomer)
Model Validation		
Ramachandran:	Core Scaffold	DARPin (Fit in Monomer)
Favored %	99.83	99.82
Allowed %	0.17	0.18
Outliers %	0	0
MolProbity score	1.21	1.51

## Data Availability

Atomic coordinates and CryoEM maps have been deposited in the Protein Data Bank (www.wwpdb.org) and EM Data Bank (www.ebi.ac.uk/emdb), respectively. PDB ID codes: 8VDZ, core scaffold; 8VE7, DARPin monomer. EMDB codes: EMD-43157, core scaffold; EMD-43158, full DARP14; EMD-43159, trimer; EMD-43167, DARPin monomer; EMD-43265 early C1 full DARP14.

## Supplementary Materials

The following supporting information can be downloaded at: https://www.mdpi.com/article/10.3390/ijms25115665/s1.
