# Supplementary material for: Mitigating the Blurring Effect of CryoEM Averaging on a Flexible and Highly Symmetric Protein Complex through Sub-Particle Reconstruction
Source: Int J Mol Sci. 2024 May 23;25(11):5665. doi: 10.3390/ijms25115665 (PMC11171969; doi:10.3390/ijms25115665)
Supplement: Supplementary file 1 [file ijms-25-05665-s001.zip › ijms-2967219-supplementary.pdf]

Supplementary Figures

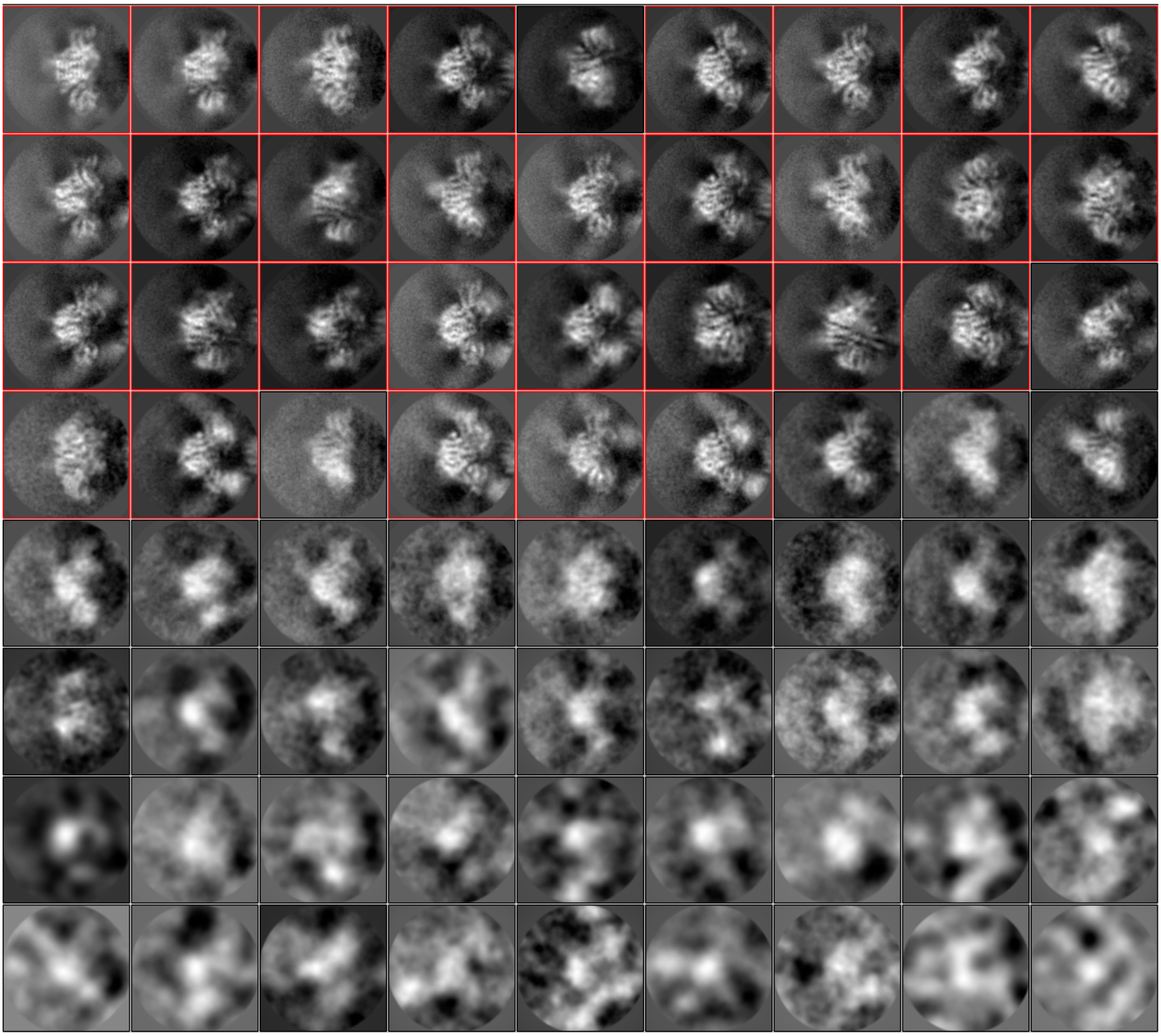

Figure S1. Example 2D classification of sub-particle trimers with chosen averages to move to the next step highlighted in Red.

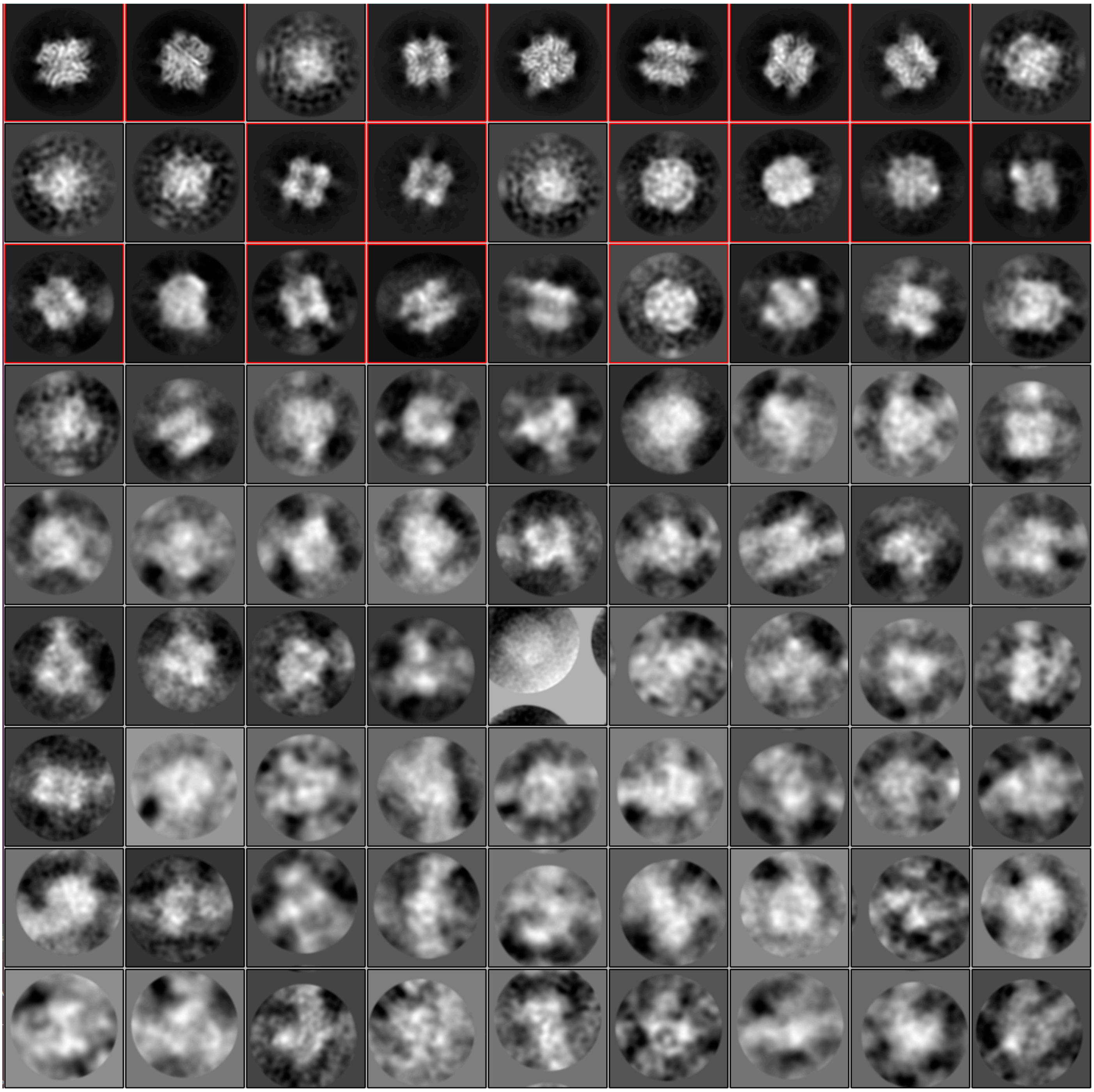

Figure S2. Example 2D classification of full DARP14 with chosen averages to move to the next step highlighted in Red.

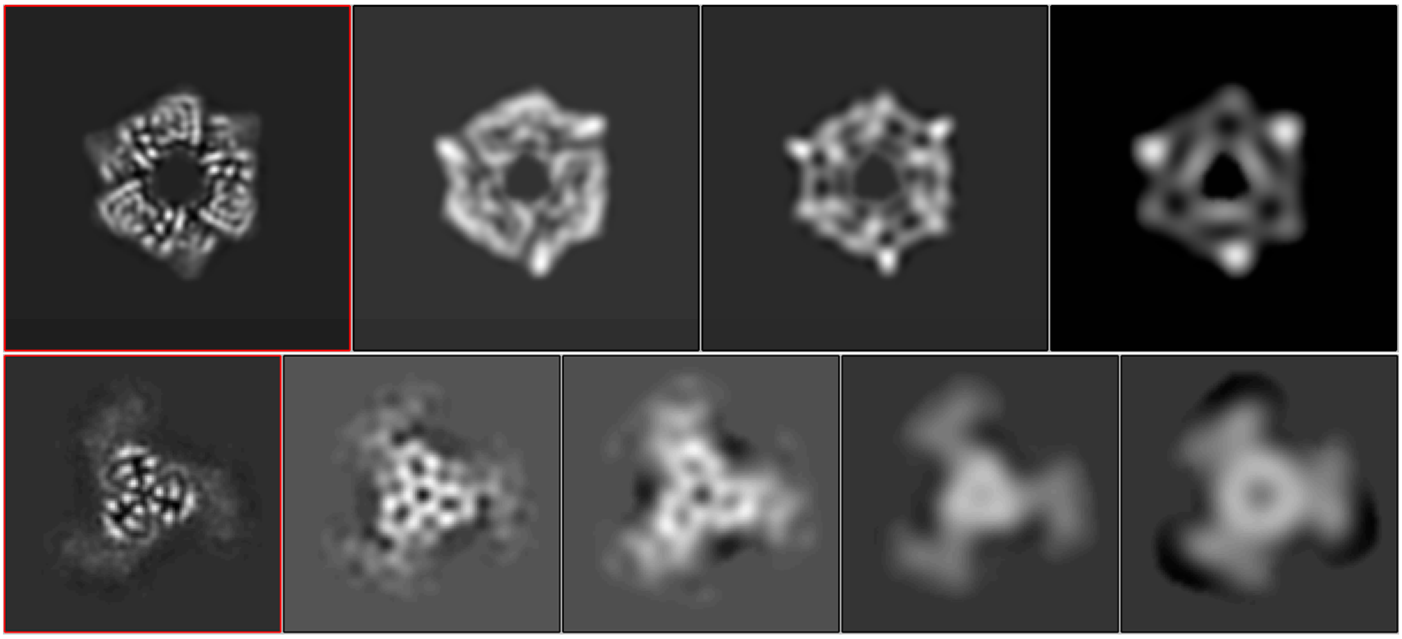

Figure S3. Example 3D classifications of core cage (top) and sub-particle trimers (bottom), with chosen classes for the next steps highlighted in red.
